# Appetite-regulating hormones, executive functions, and eating attitudes in adults with ADHD: A case-control study

**DOI:** 10.1192/j.eurpsy.2025.813

**Published:** 2025-08-26

**Authors:** Ö. Bayirli, A. Kandeğer, S. Abuşoğlu, A. E. Yorulmaz, B. Sağliyan, S. Kaya, Y. Selvİ

**Affiliations:** 1 Psychiatry; 2 Biochemistry, Selçuk University, Konya, Türkiye

## Abstract

**Introduction:**

Although the literature suggests a strong association between attention deficit hyperactivity disorder (ADHD) and obesity, the underlying mechanisms remain unclear. Eating attitudes and appetite-regulating hormones (ARH) are considered to play a role in this relationship. Recent studies have shown that ARH may be linked to executive functions, and dysregulation of these hormones may help explain the connection between ADHD and obesity.

**Objectives:**

We aimed to investigate the levels of ARH, executive functions, eating attitudes, and ADHD symptoms in adults with ADHD compared to healthy controls.

**Methods:**

The study included 44 drug-naive non-obese adults with ADHD who had no comorbid psychiatric diagnoses and 44 healthy controls matched for age, gender, education, and body mass index (BMI). All participants were diagnostically assessed using the Structured Clinical Interview for DSM-5 Disorders-Clinician Version. Also, participants completed the Adult ADHD Self-Report Scale, the Mind Excessively Wandering Scale, the Hospital Anxiety and Depression Scale, and the Eating Attitude Test (EAT). A battery of neuropsychological tests—Stroop Test (ST), Cancellation Test, Serial Digit Learning Test (SDLT), Wisconsin Card Sorting Test (WCST), and Judgment of Line Orientation Test (JLOT)—was administered. The serum samples obtained from fasting blood, after centrifugation were stored at -80°C until the time of analysis, at which point ARH levels (insulin, leptin, neuropeptide Y, orexine A, ghrelin, adiponectin) were measured using the ELISA method. The study was approved by Selçuk University Local Ethics Committee with the decision numbered 2023/328.

**Results:**

Adults with ADHD exhibited worse ADHD symptoms, disordered eating attitudes, more severe anxiety and depression, and reduced executive functioning compared to healthy controls. Although ADHD groups showed more disordered eating attitudes compared to healthy controls, there was no significant difference in ARH levels between the two groups; however, these hormone levels were associated with specific parameters from ST, SDLT, WCST, and JLOT. Linear regression analyses to identify factors associated with each ARH separately revealed significant F values, except for ghrelin, which explained a unique variance ranging from 23.5% to 36%. These results indicated that visuospatial ability was associated with each ARH levels, even after controlling for age, gender, years of education, body mass index, severity of disordered eating attitudes, and the absence of an ADHD diagnosis.

**Conclusions:**

Our findings suggest that dysregulation of ARH may associate cognitive processes related to executive functioning independent of disordered eating attitudes, BMI, and ADHD diagnosis. However, these hormones may be mediating factors in relation between ADHD and obesity, and to figure out this relation, longitudinal clinical studies with larger samples are needed.

**Disclosure of Interest:**

None Declared

